# The HortONS study. Treatment of chronic cluster headache with transcutaneous electrical nerve stimulation and occipital nerve stimulation: study protocol for a prospective, investigator-initiated, double-blinded, randomized, placebo-controlled trial

**DOI:** 10.1186/s12883-023-03435-9

**Published:** 2023-10-21

**Authors:** Ida Stisen Fogh-Andersen, Jens Christian Hedemann Sørensen, Anja Sofie Petersen, Rigmor Højland Jensen, Kaare Meier

**Affiliations:** 1 Department of Neurosurgery, Aarhus University Hospital, Palle Juul-Jensens Boulevard 165J, 8200 Aarhus, Denmark; 2 Center for Experimental Neuroscience (CENSE), Institute of Clinical Medicine, Aarhus University, Aarhus, Denmark; 3 Danish Headache Centre, Rigshospitalet-Glostrup, Copenhagen, Denmark; 4 Institute of Clinical Medicine, University of Copenhagen, Copenhagen, Denmark; 5 Department of Anesthesiology, Aarhus University Hospital, Aarhus, Denmark

**Keywords:** Chronic cluster headache, Headache, Occipital nerve stimulation, Neuromodulation, Transcutaneous electrical nerve stimulation, ONS, TENS

## Abstract

**Background:**

Chronic cluster headache (CCH) is a debilitating primary headache disorder. Occipital nerve stimulation (ONS) has shown the potential to reduce attack frequency, but the occipital paresthesia evoked by conventional (tonic) stimulation challenges a blinded comparison of active stimulation and placebo. Burst ONS offers paresthesia-free stimulation, enabling a blinded, placebo-controlled study.

Identification of a feasible preoperative test would help select the best candidates for implantation.

This study aims to explore ONS as a preventive treatment for CCH, comparing burst stimulation to tonic stimulation and placebo, and possibly identifying a potential preoperative predictor.

**Methods:**

An investigator-initiated, double-blinded, randomized, placebo-controlled trial is conducted, including 40 patients with CCH. Eligible patients complete a trial with the following elements: I) four weeks of baseline observation, II) 12 weeks of transcutaneous electrical nerve stimulation (TENS) of the occipital nerves, III) implantation of a full ONS system followed by 2 week grace period, IV) 12 weeks of blinded trial with 1:1 randomization to either placebo (deactivated ONS system) or burst (paresthesia-free) stimulation, and V) 12 weeks of tonic stimulation.

The primary outcomes are the reduction in headache attack frequency with TENS and ONS and treatment safety. Secondary outcomes are treatment efficacy of burst versus tonic ONS, the feasibility of TENS as a predictor for ONS outcome, reduction in headache pain intensity (numeric rating scale), reduction in background headache, the patient’s impression of change (PGIC), health-related quality of life (EuroQoL-5D), self-reported sleep quality, and symptoms of anxiety and depression (Hospital Anxiety and Depression Scale, HADS).

Data on headache attack characteristics are registered weekly. Data on patient-reported outcomes are assessed after each trial phase.

**Discussion:**

The study design allows a comparison between burst ONS and placebo in refractory CCH and enables a comparison of the efficacy of burst and tonic ONS. It will provide information about the effect of burst ONS and explore whether the addition of this stimulation paradigm may improve stimulation protocols.

TENS is evaluated as a feasible preoperative screening tool for ONS outcomes by comparing the effect of attack prevention of TENS and tonic ONS.

**Trial registration:**

The study is registered at Clinicaltrials.gov (trial registration number NCT05023460, registration date 07–27-2023).

## Background

### Cluster headache

Cluster headache (CH) is a primary headache disorder characterized by attacks of strictly unilateral intense intra- or periorbital pain that lasts 15–180 min and often occurs multiple times a day. These painful attacks are often accompanied by ipsilateral autonomic facial symptoms such as nasal congestion and tearing [1]. The lifetime prevalence is about 1:1000 people [2, 3], and often debuts before the age of 40, potentially reducing the ability to live a normal family life and negatively impacting the ability to work [4, 5].

The majority of patients have episodic cluster headache where the headache attacks occur in bouts of weeks to months followed by a period of remission. However, approximately 15–20% have chronic cluster headache (CCH) [6] where the patients have attacks year-round with no remission periods longer than three months [1].

The headache attacks may be shortened by the use of injectable or intranasal triptans or oxygen inhalation that can be effective abortive treatments [7, 8], whereas preventive medication such as verapamil or lithium may reduce the number and severity of attacks [9, 10]. However, patients with CCH seem to respond less to abortive treatment than patients with episodic cluster headache, and the overall effect of preventive treatment is only moderate [11]. In addition, a proportion of CCH patients are medically refractory and still suffering from frequent attacks despite relevant trials with preventive medical treatment [11, 12] either because of insufficient treatment effects or due to intolerable side effects. This leaves patients with refractory CCH without any sufficient medical treatment options.

### Occipital nerve stimulation

The International Neuromodulation Society defines therapeutic neuromodulation as “the alteration of nerve activity through targeted delivery of a stimulus, such as electrical stimulation or chemical agents, to specific neurological sites in the body” [13]. In electrical neuromodulation, the nervous system can be modulated by a weak electrical current delivered by an electrode implanted in or close to a nerve structure.

Studies have indicated that electrical stimulation of the occipital nerves can be used as a preventive treatment for refractory CCH with the potential to significantly reduce the frequency of headache attacks and—to some extent—also the pain intensity [14–19]. Occipital nerve stimulation (ONS) is an invasive but non-destructive procedure where one or two leads for electrical stimulation are permanently implanted subcutaneously across the greater occipital nerves (GON). The lead(s) is connected to an implantable pulse generator (IPG) that produces the electrical pulses for the stimulation. Depending on the surgical technique, the IPG is typically placed subcutaneously in the gluteal or sub-clavicular region.

The electrical pulses used in conventional (“tonic”) ONS evoke perceptible occipital paresthesia which challenges blinded comparison with placebo, but open-label studies find that approximately two-thirds of patients treated with ONS experience a reduction in attack frequency of 50% or more [10–15], markedly improving the patients quality of life [20]. Importantly, the therapeutic effect seems to be long-term [21, 22]. To our knowledge, only one large blinded, randomized trial on ONS for CCH has been published, exploring a dose–response relationship between ONS efficacy and stimulation intensity [23].

A novel stimulation paradigm termed burst stimulation, originally designed for spinal cord stimulation for chronic neuropathic pain, has given rise to paresthesia-free stimulation [24]. The scientific evidence on burst stimulation used for ONS is scarce, but in two small studies, it has been suggested that paresthesia-free burst stimulation may have a similar effect as tonic ONS reducing attack frequency in CCH patients [25, 26].

### Preoperative screening

Not all patients with CCH treated with ONS seem to experience sufficient if any, effect of ONS treatment [23]. Currently, no well-documented predictor for ONS outcome is known. Because of the invasive nature of the treatment, and the fact that ONS is characterized by high initial device costs, it would be favorable to have a reliable screening tool to help select the best candidates for implantation and avoid operating on patients who are unlikely to benefit from the ONS treatment.

It has been investigated if a previous good clinical response to interim treatment with prednisolone and/or a local anesthetic injected around the GON (i.e., a GON block) could predict ONS outcome, but without convincing results [27, 28]. Also, no specific clinical phenotypes that can distinguish which CCH patients are most likely treatment responders from non-responders have yet been identified.

In a small study, it has been suggested that non-invasive external transcutaneous electrical nerve stimulation (TENS) of the GON could be a possible predictor for ONS outcome [29], but it remains to be systematically explored whether TENS is a feasible preoperative screening tool.

The purpose of this study is to validate the efficacy and safety of ONS as a treatment for CCH and to explore whether burst ONS is as effective as headache attack prevention as conventional tonic ONS. In addition, we wish to investigate whether TENS of the greater occipital nerves (GON) can be used as a predictor of the effect of surgical treatment with ONS for CCH.

## Methods: Participants, interventions, and outcomes

### Study design

The study is an investigator-initiated, prospective, double-blinded, randomized, placebo-controlled trial.

**Fig. 1:**
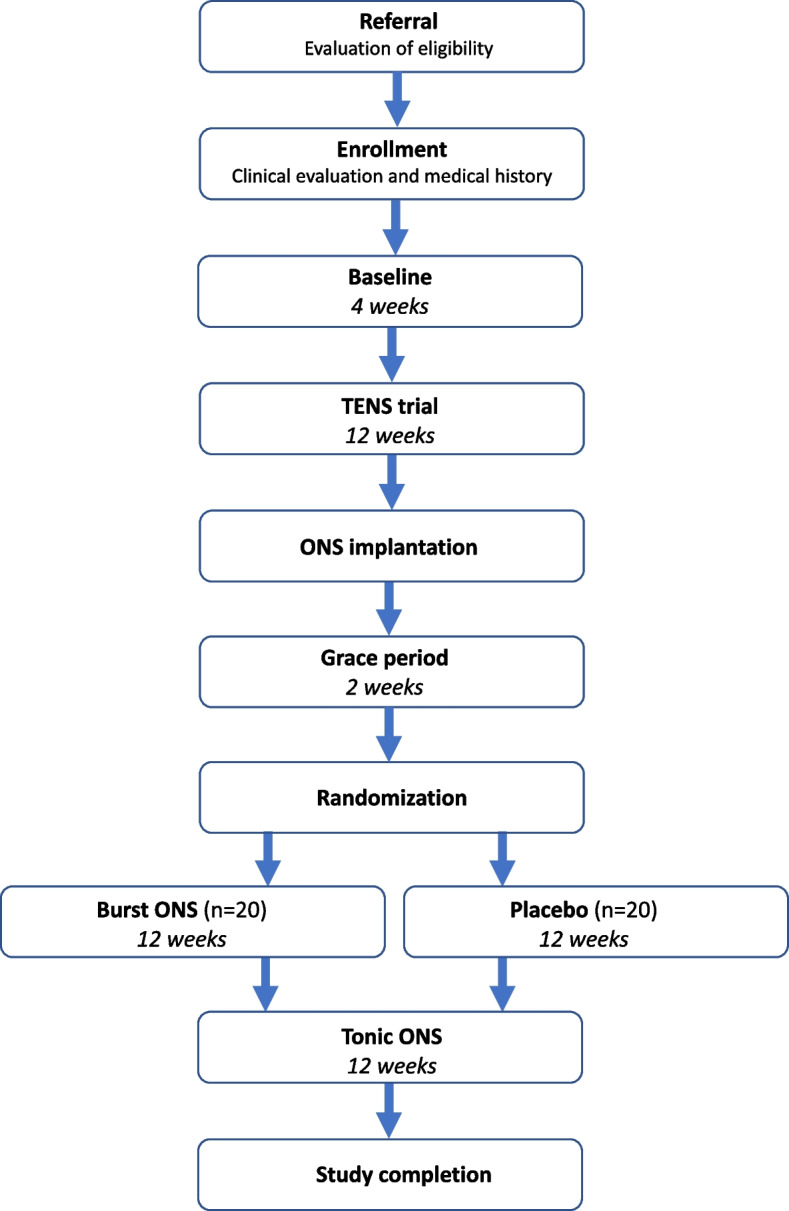
Study flowchart. TENS, transcutaneous electrical nerve stimulation. ONS, occipital nerve stimulation

After an initial baseline period, all participants are treated with TENS. Following the TENS trial all participants are implanted with an ONS system and randomized into one of two arms; one intervention arm and one placebo arm with an allocation ratio of 1:1. The participants in the intervention arm receive burst ONS (paresthesia-free stimulation). In the placebo group, the ONS system will be left turned off. In the final trial phase, all participants cross over to open-label tonic ONS (Fig. 1).

### Study setting

Patient recruitment and follow-up are carried out at the Department of Neurosurgery, Aarhus University Hospital, and the Danish Headache Centre, Rigshospitalet-Glostrup, University of Copenhagen, Denmark.

All ONS implantations and surgical follow-up visits are performed at the Department of Neurosurgery at Aarhus University Hospital, Denmark.

### Eligibility criteria

A total of 40 patients with CCH will be included in the study and have a full ONS system implanted. The diagnosis is confirmed after a thorough neurological assessment based on the criteria listed in the *International Classification of Headache Disorders, 3. Edition (ICHD-3)* [1], see Table 1 and Table 2.

**Table 1:** Diagnostic criteria for cluster headache

A. At least five attacks fulfilling criteria B-D
B. Severe or very severe unilateral orbital, supraorbital, and/or temporal pain lasting 15–180 min (when untreated)
C. Either or both of the following
1. At least one of the following symptoms or signs, ipsilateral to the headache:
• Conjunctival injection and/or lacrimation
• Nasal congestion and/or rhinorrhea
• Eyelid edema
• Forehead and facial sweating
• Miosis and/or ptosis
2. A sense of restlessness or agitation
D. Occurring with a frequency between one every other day and 8 per day
E. Not better accounted for by another ICHD-3 diagnosis

**Table 2:** Chronic cluster headache

A. Attacks fulfilling criteria A-E for Cluster headache, and criterion B below
B. Occurring without a remission period, or with remissions lasting < 3 months, for at least one year

*ICHD-3* International classification of headache disorders 3^rd^ edition

In addition to meeting the ICHD-3 criteria for CCH, participants must also comply with the study eligibility criteria listed in Table 3.

**Table 3:** Eligibility criteria

**Inclusion criteria**
• Age of 18 years and above
• Signed informed written consent
• Chronic cluster headache with ≥ 15 attacks per month
• Stable use of preventive cluster headache medication 30 days prior to study enrollment
**Exclusion criteria**
• Other ongoing neuromodulation therapies
• Current alcohol and/or drug abuse
• Known severe psychiatric disorders
• A concomitant second type of chronic primary or chronic secondary headache (e.g., chronic migraine or chronic post-traumatic headache, while chronic tension-type headache is allowed)
• Major posterior cervical surgery at C3-level and above
• Pregnancy
• Treatment with oral steroids and/or GON block 30 days before study enrollment

During the trial, the participants may continue their usual preventive and abortive headache medication, but they cannot have any dose increase of their current medication or start new preventive medication. Neither can the participants receive any interim treatment such as GON blocks (with injected steroids and/or local anesthetics) or high-dose oral prednisolone after enrollment. In this case, the participant will be withdrawn from the study. A reduction in medicine dose or discontinuation of a drug is allowed.

### Study intervention methods

#### TENS

The TENS apparatus (Perfect TENS, TensCare Ltd., Surrey UK) consists of a stimulation unit, a lead wire with two connections, and two circular 25 mm diameter electrode gel pads. The two electrode gel pads are placed bilaterally approximately at the level of the external occipital protuberance (inion) 3–4 cm from the midline where the GON has a more superficial course and where recruitment of neck muscles is avoided. Prior to electrode placement, two areas of the scalp in this site have been closely shaved with an electronic shaver to achieve a better contact surface between the skin and the electrode gel pads.

The stimulation programs used in the TENS trial are pre-set by the manufacturer. The participants can change freely between three different programs, (1) 110Hz/50μs, (2) 10Hz/200μs, and (3) 100Hz/200μs (frequency/pulse width), according to their preference. The usage pattern of the TENS equipment is individual, but participants are encouraged to use the stimulation 30 min twice a day as a minimum, and no more than 12 h in a row to avoid scalp irritation. Patients are advised to use TENS mainly as a preventive treatment, but may freely test if they achieve any abortive effect if using the stimulation during headache attacks.

#### ONS implantation

A cylindrical 8-contact lead (SC-2366–70 Linear 3–6 Percutaneous Lead, Boston Scientific, Marlborough MA, USA) is implanted subcutaneously in the occipital region via a bent 14G Touhy cannula inserted through two incisions over the external occipital protuberance (inion) and behind the mastoid, respectively. The lead is placed transversely approximately one cm above the level of the inion, aiming to cover both the right and left GON. The lead is fixed to the pericranium using an anchor (Injex Bumpy Anchor 97,791, Medtronic, Minneapolis MN, USA), tunneled subcutaneously along the neck, and connected to an IPG (SC-1416 WaveWriter Alpha Prime 16, Boston Scientific) placed subcutaneously in the right infraclavicular region. The implantations are performed as a one-stage sleep/awake procedure with an on-table test stimulation of the implanted lead in order to secure the correct positioning of the lead leading to satisfactory bilateral occipital paresthesia. If necessary, the position of the lead is adjusted based on the participants' verbal feedback on the localization of the evoked paresthesia.

#### Burst ONS

Burst stimulation is a stimulation paradigm devised to mimic the natural firing pattern of neurons by delivering electrical impulses in bursts (short trains separated by a gap). These impulses are delivered in sub-sensory amplitudes resulting in a paresthesia-free stimulation. The parameters for burst ONS (“MicroBurst”, Boston Scientific) are set with an intra-burst frequency of 450 Hz (six pulses), an inter-burst frequency of 40 Hz, and a pulse width of 300 μs [30]. Stimulation amplitude is set to 50% of the detection threshold.

Participants allocated to the burst ONS group receive this stimulation continuously throughout the 12-week blinded trial phase.

#### Placebo

Placebo treatment is performed by leaving the ONS system turned off after the initial programming session throughout the 12-week blinded trial phase.

#### Tonic ONS

In conventional tonic stimulation, the electrical impulses are delivered as regular biphasic square waves. All participants cross over to open-label tonic ONS as the final phase of the trial regardless of allocation group during the blinded part of the trial. A personal remote control allows the participants to change between three different pre-set stimulation programs with frequencies of 10, 30, and 100 Hz, respectively. Pulse width (250–500 µs) and configuration (polarity and activation) of the eight contacts on the linear lead are adjusted to achieve the best possible occipital paresthesia. The participants’ individual perception and comfort thresholds determine the amplitudes and can be controlled using the remote control. The participants are encouraged to use the stimulation continuously but are allowed intermittent use if preferred.

#### Post-procedural care

After ONS implantation, patients are admitted for one night and discharged the following day after a clinical control. To achieve the best possible conditions for programming the ONS system before randomization and blinding, there is a 14-day grace period to ensure the healing of the surgical wounds and reduced tissue swelling. To minimize the risk of postoperative infection, ten days of peroral antibiotics (dicloxacillin 1000 mg three times a day) are prescribed.

### Outcome measures

**Fig. 2:**
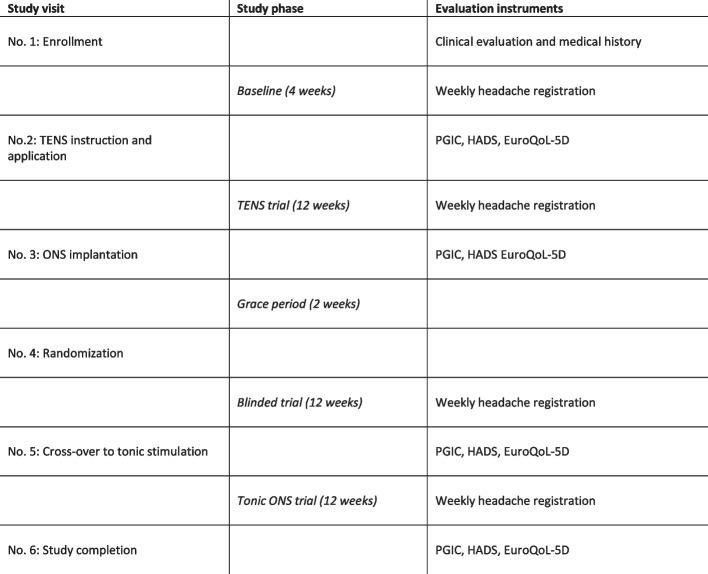
Outcome parameters in correlation to trial phases and study visits. TENS, transcutaneous electrical nerve stimulation. ONS, occipital nerve stimulation. PGIC, patient's global impression of change. HADS, hospital anxiety and depression scale. EuroQoL-5D, European Quality of Life – 5 Dimensions

The response to the different intervention methods is continuously registered by participants filling in a weekly electronic headache registration. Questionnaires on health-related quality of life, self-perceived treatment effect, and symptoms of anxiety and depression prior to every follow-up visit (Fig. 2).

All questionnaires are electronic and are entered via a personal link sent automatically by e-mail. If the headache registrations and questionnaires are not completed within 24 h, an e-mail reminder is generated.

The data collected during each trial phase will be compared to the baseline and analyzed in order to evaluate the study endpoints listed in Table 4.

**Table 4:** Study endpoints

**Primary endpoints:**
1. 30% reduction in attack frequency with TENS and ONS
2. Safety in ONS and TENS treatment
**Secondary endpoints**
1. Participant’s assessment of treatment effect evaluated by PGIC-score
2. Treatment efficacy of burst versus tonic ONS as a preventive treatment for chronic cluster headache
3. Feasibility of TENS as a predictor for the outcome of ONS treatment
4. 30% reduction in pain intensity
5. Quality of life as evaluated with the EuroQoL-5D questionnaire
6. Reduction in background headache
7. Self-reported sleep quality
8. Symptoms of anxiety and depression as evaluated by HADS

*TENS* Transcutaneous electrical nerve stimulation, *ONS* Occipital nerve stimulation, *PGIC* Patient's global impression of change, *HADS* Hospital anxiety, and depression scale, *EuroQoL-5D* European Quality of Life – 5 Dimensions

### Headache registration

Participants log information about their headache attacks the past week; number of attacks, mean duration of attacks (in minutes), and severity of attacks on a 0–10 numeric rating scale (NRS, 0 is no pain, 10 is the worst pain imaginable) [31]. In addition, the use of abortive medication and/or oxygen inhalation is registered, as well as the presence of background headache, autonomic facial symptoms, and the participants’ self-reported sleep quality on a four-point Likert scale.

During the TENS- and tonic ONS trial phase, the weekly headache registration will have supplementary questions regarding the use of stimulation; usage pattern (continuous, intermittent, no stimulation), and preferred stimulation program(s).

### Patient’s Global Impression of Change (PGIC)

PGIC is a single, self-administered tool asking respondents to rate how their condition has changed since a certain point in time [32].

In this study, PGIC is used to record the participants’ self-reported perception of the effect of a given treatment (TENS, burst ONS, and tonic ONS) based on a seven-point scale ranging compared to before the treatment was initiated (baseline).

### European Quality of Life – 5 Dimensions (EuroQoL-5D)

EuroQoL-5D is a standardized tool for examining the health-related quality of life, and consists of a descriptive part with five questions about mobility, personal care, usual activities, pain, and anxiety/depression. In addition, the questionnaire also contains the EQ-VAS (visual analog scale), which measures the participant's self-assessed state of health on a 0–100 scale from "worst imaginable" to "best imaginable" [33].

In this study, the participants fill out the EuroQoL-5D questionnaire at the end of each trial phase (baseline, TENS trial, blinded trial, and tonic ONS trial).

### The Hospital Anxiety and Depression Scale (HADS)

A simple, validated tool for evaluating patients' risk of anxiety and/or depression primarily focusing on non-physical symptoms [34]. The questionnaire comprises seven questions for depression (HADS-D subscale) and seven questions for anxiety (HADS-A subscale). Scoring for each item ranges from zero to three, with a total subscale score of ≥ 8 points out of a possible 21 denoting considerable symptoms of anxiety or depression. HADS is useful both for screening and for monitoring the progression or resolution of psychological symptoms.

In this study, the participants fill out the HADS questionnaire at the end of each trial phase (baseline, TENS trial, blinded trial, and tonic ONS trial).

### Participant information

All participants sign an informed consent before enrolment and are given both oral and written information before consenting. This includes information about the purpose of the project, potential risks and side effects, and the right to terminate the trial at any given time without consequences for present or future medical treatment. It is ensured that the participants fully understand the information given by the principal investigator at the Department of Neurosurgery, Aarhus University Hospital, or site investigators at the Danish Headache Centre. Participants are encouraged to bring a next of kin to the consultation.

Before enrolling the participants are informed of, and consent to, the fact that they are blinded to the type of treatment they receive in the randomized trial phase.

The written information is published as a pamphlet explaining all information in Danish layman’s language. The content and wording of the written participant information are approved by the Central Denmark Region Committee on Health Research Ethics.

The pamphlet will clearly state the potential risks and side effects of all procedures involved in the study.

1. TENS: Perceptible occipital paresthesia during stimulation. Risk of irritation of the skin covered by the gel electrodes.
2. ONS-implantation: Risk of infection and hematoma. Lesion of nerve trunks and blood vessels during placement and tunneling of the lead. Risk of pneumothorax when tunneling the lead. Post-operative pain.
3. ONS treatment: Perceptible occipital paresthesia during tonic stimulation. Pain and/or discomfort at IPG site. Risk of lead migration or fracture. Preterm battery depletion.

### Sample size

Based on our previous experience with ONS treatment [26], we estimate that 50% of the participants who receive active treatment with burst ONS will reach the primary endpoint of a 30% reduction in attack frequency. A blinded trial with burst ONS and placebo has to our knowledge not been carried out earlier, but we hypothesize that no more than 10% in the placebo group will meet the primary endpoint [35]. The sample size is calculated using Fisher’s Exact test based on the above-mentioned assumptions and with 80% power (two-tailed) and a α = 0.05, a total of 40 participants (20 in each allocation group) are needed to be enrolled in the study and an ONS-system implanted.

Each enrolled participant contributes their data regardless of whether they complete the project or not. In case of participants excluded after enrollment, the reason for exclusion will be described.

### Patient recruitment

Recruitment commenced in September 2021 and will continue until the sample size is reached. Recruitment is expected to be completed at the end of 2023.

Participants are recruited from all over Denmark and are primarily referred from regional headache clinics and the Danish Headache Center, a tertiary headache referral center.

## Methods: assignment of interventions

### Randomization and blinding

After the 14-day grace period following ONS implantation, the ONS systems will be programmed and the participants will be allocated 1:1 in the two study groups (burst ONS or placebo). The allocation is conducted by block randomization with blocks of random size between four and eight, using a specially designed automated electronic randomization tool. The allocation tables for the randomization are inaccessible to the investigators and other staff involved in the project.

The randomization is performed by the implanter or the project nurse who is responsible for the programming of the ONS systems. These personnel are unblinded throughout the entire trial and are therefore able to handle any ONS-related events during the blinded trial phase without mitigating the blinding. Unblinded personnel will not have any role in data collection, management, and analysis.

The participants are blinded to their allocation group. The principal investigator and other study personnel who perform the evaluation of outcome parameters, are blinded to the allocation group as well, and will not have access to the randomization tool in the project database. Blinding is maintained until all participants have completed the final trial phase.

The IPGs used in the study are non-rechargeable units to ensure that a lack of need for recharging does not unblind the participants. In addition to this, to avoid accidental unblinding, the participants will not have the remote controls to the ONS system during the blinded trial phase, since the display would reveal if stimulation is on. Importantly, when treated with burst stimulation, participants should not have any sensation of stimulation and, hence will not need to adjust the stimulation amplitude. The postoperative 14-day grace period ensures accurate programming, which in our experience limits the need for stimulation adjustments within the 12 weeks of blinded trial to an absolute minimum. If needed, patients can directly contact the principal investigator. Should the participants get a sensation of stimulation during the blinded trial phase, unblinded personnel can adjust the stimulation settings if necessary.

When crossing over to the open-label tonic ONS trial phase, all participants are equipped with a remote control.

## Methods: data collection, management, and analysis

### Data collection and management

The study data is collected in compliance with the General Data Protection Regulation (GDPR). The paper consent forms are stored in a locked container. All other forms and questionnaires are electronic and are collected in the Research Electronic Data Capture (REDCap) database system hosted at Aarhus University [36, 37] in a dedicated project database.

Acquisition, storage, and analysis of the data are authorized by the Central Denmark Region (permit number 1–16-02–577-20).

### Statistical analysis

Statistical analysis will be conducted by the principal investigator and a statistician blinded for treatment allocation only after follow-up for all participants is completed. No interim analysis will be performed.

Characteristics of the patients will be presented using descriptive statistics to assess if balanced groups were obtained after randomization. The primary analysis will be conducted according to the intention-to-treat principle to avoid introducing bias. In a secondary analysis, a per-protocol analysis will be performed.

The primary outcome is a 30% reduction in attack frequency with TENS and ONS treatment compared to baseline. Relative change in attack frequency (in percent) will be analyzed using the Wilcoxon signed-rank test for paired variables, comparing the baseline data with data from the TENS trial and the trial with burst and tonic ONS, respectively. Because the changes in attack frequency are expectedly skewed [23], a non-parametric test will be applied. In cases with a minimum 30% reduction in attack frequency, sub-analyses will be made to group the treatment responders after relevant cutoffs.

Analysis of the secondary outcomes will be conducted according to the same principle, comparing data from each of the trial phases to baseline using a parametric or non-parametric test for paired variables, depending on the data distribution.

The feasibility of TENS as a predictor for ONS outcome will be evaluated by determining the sensitivity and specificity and calculating the predictive values.

Statistical data analysis will be performed using Stata 17.0 (StataCorp, College Station, USA-TX).

## Methods: monitoring

### Data monitoring

The principal investigator is responsible for maintaining the participant’s anonymity. The participants are informed of and consent to the research team accessing their medical records and registering personal health information of relevance to the study and study procedures, such as comorbidities and medication. The REDCap database can be accessed only by users specially assigned to the project, and login requires a personal username with two-factor authentication. All access to the database is fully logged and all actions are audited and can be traced. The principal investigator has full access to the database, except the randomization module which is fully shielded from all personnel except the implanters and device programmers.

### Adverse events

The principal investigator is responsible for continuously registering adverse events and side effects of any kind related to the TENS and ONS treatment. Data on all adverse events and side effects are registered and logged in the project-specific REDCap database. All side effects and adverse events are reported to the Central Denmark Region Committee on Health Research Ethics once a year, and collectively when the project is completed. Serious adverse events will be reported immediately to the Central Denmark Region Committee on Health Research Ethics.

In the blinded trial phase (burst ONS vs. placebo), none of the participants have access to the remote control for their ONS system, as this could potentially reveal the randomization of the participant. A clinical control after the postoperative grace period of 14 days is scheduled to ensure optimal conditions for program optimization. Should the participants suspect a problem related to the ONS system, direct contact can be made with the principal investigator, and the participant will then be seen by project staff who are trained in programming the system and who are unblinded for the randomization group. Where necessary, a clinical evaluation will be made by the ONS implanters at the Department of Neurosurgery, Aarhus University Hospital.

## Discussion

The effect of ONS on CCH has primarily been evaluated in case series and uncontrolled open-label studies [14–19], aside from a single large dose–response RCT [23]. In the 2023 European Academy of Neurology Guidelines on Cluster Headache [38] there is no recommendation for ONS due to the very low level of evidence. To our knowledge, the study presented here is the first randomized, blinded, placebo-controlled trial on ONS treatment for CCH; when completed, it should constitute a significant contribution to the evidence in the field.

Although little is known about burst stimulation for ONS, there are indications that this type of stimulation also has a preventive treatment effect in CCH patients. Because the participants do not perceive any sensation of stimulation, it enables the conduction of a blinded trial with burst ONS as the active comparator to an inactive placebo treatment.

Burst stimulation was introduced in 2010 and later marketed by neuromodulation device manufacturer Abbott as BurstDR. Subsequently, other manufacturers of neuromodulation systems have developed similar waveforms marketed under other burst labels. A difference in mechanism of action has been claimed [39], however, in this study we do not distinguish between the different variations of the burst waveforms.

The study design allows a comparison between burst ONS and placebo and enables a head-to-head comparison of the efficacy of burst and tonic ONS. In addition, by comparing the effect of treatment with TENS and tonic ONS, respectively, it is possible to evaluate whether the effect of TENS can be used as a feasible preoperative screening tool for ONS outcomes and can be used to select the most eligible candidates for implantation.

There is no official consensus about optimal stimulation settings in ONS for CCH. The study will provide important information about the efficacy of burst ONS and explore whether the addition of this stimulation paradigm might help improve future stimulation protocols.

## Data Availability

All data is stored in the REDCap database system which can only be accessed by the primary investigator and study group. The full dataset will not be made available as further analyses may be performed including long-term follow-up analyses.

## Abbreviations

CCH: Chronic cluster headache
GON: Greater occipital nerve
HADS: Hospital anxiety and depression scale
IPG: Implantable pulse generator
NRS: Numerical rating scale
ONS: Occipital nerve stimulation
PGIC: Patient's global impression of change
TENS: Transcutaneous electrical nerve stimulation
